# The impact of NPK fertilizer on growth and nutrient accumulation in juniper (*Juniperus procera*) trees grown on fire-damaged and intact soils

**DOI:** 10.1371/journal.pone.0262685

**Published:** 2022-01-27

**Authors:** Ahlam Khalofah, Hamed A. Ghramh, Rahmah N. Al-Qthanin, Boullbaba L’taief

**Affiliations:** 1 Biology Department, Faculty of Science, King Khalid University, Abha, Saudi Arabia; 2 Research Center for Advanced Materials Science (RCAMS), King Khalid University, Abha, Saudi Arabia; 3 Unit of Bee Research and Honey Production, Faculty of Science, King Khalid University, Abha, Saudi Arabia; 4 Prince Sultan Bin-Abdul-Aziz Center for Environment and Tourism Studies and Researches, King Khalid University, Abha, Saudi Arabia; 5 Laboratory of Agronomic Science and Technology (LR16INRAT03), National Institute of Agronomic Research of Tunisia (INRAT), University of Carthage, Tunis, Tunisia; Harran University: Harran Universitesi, TURKEY

## Abstract

Wildfires significantly alter soil properties and result in vegetation shifts; therefore, rapid reforestation activities are needed in the forests affected by wildfires. The decreased nutrient in the soil is the obvious effect of wildfires; however, little is known about the reforestation of Juniper *(Juniperus procera*) forests with application of NPK fertilizers. Juniper forests are common in Asir and Taif regions of Saudi Arabia and vulnerable to wildfires; thus, reforestation is needed after the onset of fires. This study assessed the impact of different doses of organic NPK fertilizer (0, 5 and 10 g/L) on growth and nutrient accumulation of Juniper trees grown on fire-damaged and intact soils. Data relating to tree height, number of leaves per plant, fresh and dry biomass accumulation in shoot and root, chlorophyll contents and uptake of N, P, K, and Na were recorded. Individual and interactive effects of soil types and fertilizer doses significantly altered all measured traits with minor exceptions. Overall, higher values of the measured traits were recorded for intact soil and 10 g/L fertilize dose. The increasing fertilizer doses improved the growth and nutrient acquisition and application of 10 g/L fertilizer on intact soil recorded the highest values of growth traits. Juniper trees grown on fire-damaged soil accumulated higher amount of nitrogen than intact soil. Similarly, the trees grown on intact soil accumulated lower amount of Na and maintained comparable K/Na ratio to intact soil. It is concluded that supplying 10 g/L fertilizer could improve the establishment of Juniper trees on fire-damaged soil. Therefore, organic fertilizer can be used to improve the reforestation of wildfire-affected Juniper forests in the Asir province.

## Introduction

Forests are of significance importance globally due to their valuable ecosystem services and provision of quality wood. However, the ecosystem services are still far below than the potential of intact forest ecosystems because of several reasons. Forests protect environment and watersheds, stabilize land, control desertification, fix sand dunes, restore soil fertility, and mitigate microclimatic changes around the world [1, 2]. The other valuable services include provision of timber wood and non-wood products, as well as grazing place for animals. Furthermore, forests serve as an important habitat for wildlife and native vegetation. Livelihoods of numerous global communities are derived from various goods and services provided by forest ecosystems. Besides, significant amount of global carbon stock is stored in forest ecosystems [1, 3, 4].

Globally forest ecosystems are exposed to wildfires. Wildfires naturally occur in the forest ecosystems and their frequency increases with increasing human development [5]. Moreover, global warming is also increasing the frequency and intensity of wildfires due to rising temperature [6]. Soil and associated biological systems are significantly altered by wildfires. The extent of alterations in the biological systems and soil properties is dependent on the frequency and severity of the wildfires [7]. Increasing temperature coupled with humid environment results in the occurrence of wildfires at different spatial scales. Wildfires can be originated from natural or anthropogenic sources. Rapidly changing climate would increase the occurrence of wildfires due to increased temperature, which would burn above-ground biomass, alter soil and vegetation, carbon dynamics and composition of the species [8, 9]. Flora, fauna, and soil characteristics have been altered in Asian and European regions by wildfires [8, 9]. The risk of wildfires is often reduced through prescribed burning [10]. Vegetation loss, shrubby and grassland vegetation, and soil degradation are the main outcomes of wildfires [7, 11–13].

The predicted climate changes would increase the occurrence of wildfires in a warming world [14–16], particularly in highly productive areas [17]. The increased fires will burn plenty of vegetation resulting in severe negative ecological effects [15, 18]. The burning would result in high soil erosion, adverse environmental conditions, and extensive vegetation burning/shifts. These all factors would result in failed regeneration events [19–21]. Therefore, sound ways to improve the growth of tree species on fire-damaged soils should be established.

Wildfires are both beneficial and harmful for forest ecosystems depending on their severity and intensity of return. Wildfires maintain diversity and stability of forest ecosystems and affect numerous soil properties [22]. Soil organic matter, macro and micro-nutrients, soil texture, color, pH, and soil biota are altered by forest fire [12]. Combustion of litter and organic matter during low intensity fires results in increased availability of plant nutrients. However, high intensity fires result in complete loss of soil organic matter, volatilization of nutrients and death of microbes [22]. The increased availability of nutrients due to low intensity fires results in rapid recovery of the forests, whereas recovery under high intensity fires takes longer time and requires concrete efforts. Supplying plant nutrients to the forests affected by high intensity fires could accelerate their recovery. However, different forest species require varying amounts of nutrients. Therefore, determining optimum nutrient requirements for species could help in their rapid recovery.

Nitrogen (N), phosphorus (P) and potassium (K) are primary nutrients required by the plants for proper growth and development. Growth and development of plants is negatively affected by deficiency of any of these nutrients during the life cycle. Nitrogen plays a key role chlorophyll synthesis and subsequently in photosynthesis [23]. Nitrogen plays an important role in vegetative growth of plants; thus, should remain available during vegetative stage [24]. The P increases cell division and stimulates root growth and flowering [25]. The P is found in the plant parts having high metabolism and rapid cell division; therefore, plays a role in the storage and transfer of energy released during photosynthesis and its deficiency delays plant maturity [26]. Rational/artificial fertilization could improve soil nutrient status which would increase crop productivity and alter C and N input from crop residues [27]. The fertilization also influences soil C and N accumulation through changing soil structure, soil organic C and N component [28], and soil aggregation [27]. However, optimum amount of these nutrients should be determined prior to their application.

The Juniperus genus comprises of 60 species with the widest distribution in northern hemisphere excluding *Juniperus procera* Hochst. ex. Endl. which is frequently observed in southern hemisphere [2]. Juniperus ranks 3^rd^ in in terms of numbers among the conifers [29]. *Juniperus procera* can grow both in northern and southern hemispheres [2]. It can reach to 40 m height and a 3 m diameter [30]. It is distributed in Asir mountains in Saudi Arabia apart from some mixed populations around Taif. Trees are few meters tall in slopy areas, whereas reach 10–15 m height in plain areas [3, 4, 31]. The *J*. *procera* communities are observed between 2000 and 3000 m altitude [32]. Juniperus have a narrow genetic diversity, small population size, and low population density [33, 34]. Sudden die-off of Juniperus could be an outcome of drought stress caused by global warming. In Saudi Arabia, populations of *J*. *procera* in Asir Mountains are more severely affected by the dieback incidence than *J*. *phoenicea* in Hijaz Mountains. However, the most problem faced by *J*. *procera* forests in the Asir province is wildfires, which result in the burning of trees. Reforestation of the wildfire-affected forests is necessary to maintain the ecosystem services. However, the impact of fertilizer addition on the growth of *J*. *procera* trees grown on fire-damaged and intact soils has not been tested.

This study assessed the effect of organic fertilizer (NPK 20-20-20) on the growth and nutrient uptake of Juniper plants grown in fire-damaged and intact soils. It was hypothesized that increased dose of NPK fertilizer will increase the growth and nutrient uptake of trees compared to low dose. Furthermore, it was hypothesized that trees would have better growth and nutrient accumulation on intact soil compared to fire-damaged soil. The results will help to restore the forest areas affected by wildfires in the Asir province of Saudi Arabia.

## Materials and methods

### Experimental site

The current experiments were conducted at Research Center of Advanced Materials (RCAMS), King Khalid University (KKU), Saudi Arabia during 2020–2021.

### Experimental soils and species

Two different soils, i.e., fire-damaged (S1) and intact (not damaged by the fire) (S2) were collected from the forests of mountain Ghulamah in the Asir region (Tanomah). Soil samples (three from each fire-damaged and intact soil) were collected from 0–30 cm soil depth and physicochemical properties were determined. The samples were air-dried and passed through 2-mm sieve. Organic matter was determined by Walkley and Black method [35]. Particle size distribution (texture) was determined by hydrometer method in a sedimentation cylinder using sodium hexametaphosphate as the dispersing agent [36]. The pH and electrical conductivity (EC) were measured in a saturated soil paste [37]. Total P and K were analyzed by following Olsen [38] and Carson [39], respectively. Total N in the soil was determined by Kjeldahl method [40]. The soil properties of both soils are given in Table 1. Juniper *(Juniperus procera*) trees were obtained from the Ministry of Agriculture in the Asir region, Abha. The trees were 15 cm tall upon collection from the Ministry.

**Table 1:** Soil properties, carbon and nitrogen stock in fire-damaged and intact soils used in the study at the time of initiation of the experiment.

Soil property	Fire-damaged soil	Intact soil
**Sand (%)**	51.21 ± 0.56	50.90 ± 0.72
**Silt (%)**	34.12 ± 0.41	32.70 ± 0.62
**Clay (%)**	14.67 ± 0.48	17.40 ± 0.45
**Bulk density (gm** ^**−3**^ **)**	1.45 ± 0.12	1.47 ± 0.11
**pH**	8.13 ± 0.09	8.01 ± 0.19
**EC (dS m** ^**−1**^ **)**	0.21 ± 0.04	0.24 ± 0.03
**Available N (kg ha** ^**−1**^ **)**	188.45 ± 10.21	225.91 ± 11.23
**Available P (kg ha** ^**−1**^ **)**	14.45 ± 1.21	23.41 ± 1.15
**Available K (kg ha** ^**−1**^ **)**	167.89 ± 12.45	233.23 ± 11.32
**Carbon stock (t ha** ^**−1**^ **)**	14.45 ± 1.94	23.98 ± 2.32
**Nitrogen stock (t ha** ^**−1**^ **)**	1.46 ± 0.03	2.21 ± 0.08

### Experimental procedures

Juniper trees were transferred to plastic pots (25 cm diameter and 25 cm height) perforated from the bottom, filled equal amounts of soil (either damaged or undamaged soil) and peatmoss in a 1:1 ratio. The pots were placed in the greenhouse and the temperature was set to 25 ± 2 °C. All pots were irrigated 3 times a week with 400 ml water for 1 month. The trees were treated with fertilizer NPK (20-20-20) after one month with three concentrations (0, 5 and 10 g/liter) by foliar spray three times a week for 90 days.

The pots were divided into six groups, i.e., intact soil with three NPK concentrations and fire-damaged soil with three NPK concentrations. The NPK treatment continued for 90 days and NPK was foliar sprayed three times a week. At the end of the experiment, the trees were removed from the pots and washed to remove the suspended soil/debris prior to morphological and physiological measurements (photosynthetic pigments, relative water content (RWC) and concentration of N, K, P, and Na.

### Experimental design

The experiment was conducted according to randomized complete block design with factorial arrangements. Soil types were kept in main plots, whereas NPK concentrations were randomized in sub plots. Each treatment has three replications and each replication had two pots.

### Data collection

Growth traits were recorded 120 days of after the initiation of experiment (30 days of irrigation for plant establishment and 90 days of fertilizer treatment). The measurements included tree height (cm), number of leaves (leaves/tree). Fresh and dry weight of shoot and root (g) was recorded by weighing the freshly harvested and dried root and shoot samples.

For measuring photosynthetic pigments, 0.2 g fresh leaf sample was digested with 80% acetone and homogenized. The homogenized sample was filtered, and the resulting filtrates was raised to 25 mL by adding 80% acetone in each sample. The absorbance of the filtrates was determined at various wavelengths (663, 645, and 480 nm) by using an UV-1900 BMS (Germany) spectrophotometer. The chlorophylls (a and b), and total chlorophyll (total chls), were determine and expressed in mg/g FW following Arnon [41] formula.


$$\begin{array}{c} \text{Chl}\;\text{a}=\left[\left(12.7\times \text{OD}\;663\right)-\left(2.69\times \text{OD}\;645\right)\right]\times \text{V}/1000\times \text{W}.\\ \text{Chl}.\;\text{b}=\left[\left(22.9\times \text{OD}\;645\right)-\left(4.68\times \text{OD}\;663\right)\right]\times \text{V}/1000\times \text{W}.\\ \text{Total}\;\text{chls}=\left[\left(20.2\times \text{OD}\;645\right)-\left(8.02\times \text{OD}\;663\right)\right]\times \text{V}/1000\times \text{W}. \end{array}$$

Relative water contents (RWC) were measurement according Farooq et al. [42]. The leaves were weighed fresh, then kept in distilled water for 24 hours and again weighed to record turgid weight. The turgid leaves were dried in an oven and weighed to record dry weight. The RWC were measured according to the formula given by Cornic [42].


$$\text{RWC}=\left[\left(\text{FW}-\text{DW}\right)/\left(\text{TW}-\text{DW}\right)\right]\times 100$$

Here, FW = fresh weight, DW = dry weight and TW = turgid weight.

The N contents were measured according to micro-Kjeldahl method described in the AOAC [40]. The molybdenum-reduced molybdophosphoric blue color method in sulphuric acid (with reduction to exclude arsenate) was used to determine P content. Sulphomolybdic acid (molybdenum blue), diluted sulphomolybdic acid, and 8% (w/v) sodium bisulphite-H_2_SO_4_ solution were used as reagents. The K and Na contents were determined using 0.2 g of dried leaves digested with sulphuric acid in the presence of H_2_O_2_ [43]. Total leaf contents of Na and K contents were measured using Flame Spectrophotometry.

### Statical analysis

The collected data were tested for normal distribution [44] which resulted that all measured variables were normally distributed. Therefore, the original data were used in statistical analysis. Two-way analysis of variance (ANOVA) was used to test the significance in the data [45]. The means were compared with least significant difference test where ANOVA indicated significant differences. The analysis was conducted on SPSS V.21 [46]. The minimal dataset used in the analysis is given as S1 File.

## Results

The individual and interactive effects of soil types and NPK concentrations significantly altered tree height, number of leaves per plant, stem and root fresh and dry weights and relative water contents of *J*. *procera* tree with some exceptions (Table 2). Soil type had the only non-significant impact on relative water contents, whereas the rest of the traits significantly affected all the measured growth traits during the study (Table 2).

**Table 2:** Analysis of variance of different growth traits of *Juniperus procera* trees as affected by different soil types and NPK concentrations.

Source of variation	DF	Sum of squares	Mean squares	F value	P value
**Tree height**					
**Soil Type (S)**	1	202.74	202.74	518.22	< 0.0001*
**Nitrogen doses (N)**	2	97.94	48.97	125.17	< 0.0001*
**S × N**	2	10.72	5.36	13.70	0.0008*
**Number of leaves per plant**					
**Soil Type (S)**	1	40.38	40.38	72.12	< 0.0001*
**Nitrogen doses (N)**	2	446.31	223.16	398.53	< 0.0001*
**S × N**	2	6.46	3.23	5.77	0.0176*
**Stem fresh weight**					
**Soil Type (S)**	1	299.23	299.23	523.50	< 0.0001*
**Nitrogen doses (N)**	2	486.56	243.28	425.62	< 0.0001*
**S × N**	2	12.15	6.08	10.63	0.0022*
**Root fresh weight**					
**Soil Type (S)**	1	239.22	239.22	546.36	< 0.0001*
**Nitrogen doses (N)**	2	246.71	123.36	281.73	< 0.0001*
**S × N**	2	27.33	13.67	31.21	< 0.0001*
**Stem dry weight**					
**Soil Type (S)**	1	135.85	135.85	761.25	< 0.0001*
**Nitrogen doses (N)**	2	255.00	127.50	714.48	< 0.0001*
**S × N**	2	20.71	10.35	58.02	< 0.0001*
**Root dry weight**					
**Soil Type (S)**	1	86.51	86.51	617.70	< 0.0001*
**Nitrogen doses (N)**	2	218.58	109.29	780.39	< 0.0001*
**S × N**	2	24.20	12.10	86.40	< 0.0001*
**Relative water contents**					
**Soil Type (S)**	1	0.01	0.01	0.02	0.8771^NS^
**Nitrogen doses (N)**	2	83.37	41.69	111.32	< 0.0001*
**S × N**	2	13.23	6.62	17.67	0.0003*

NS = non-significant,

* = significant

Regarding the individual effects of soil types, the higher values of tree height, number of leaves per plant, and fresh and dry weights of stem and root were recorded for the trees grown on intact soil compared with fire-damaged soil. Nonetheless, relative water contents were not altered by different soil types (Table 3).

**Table 3:** The influence of different NPK doses on growth and biomass accumulation of juniper tree grown on two different soil types.

	TH (cm)	N_leaves_ (plant^-1^)	SFW (g)	RFW (g)	SDW (g)	RDW (g)	RWC (%)
**Soil types**							
**Fire damaged**	33.30 b	52.50 b	26.68 b	14.84 b	13.29 b	7.89 b	86.45 ^NS^
**Non-damaged**	40.02 a	55.49 a	34.83 a	22.13 a	18.78 a	12.28 a	86.49
**LSD 0.05**	**3.45**	**2.12**	**3.45**	**4.58**	**2.23**	**4.19**	**NS**
**Nitrogen doses**							
**0**	33.79 c	48.11 c	24.42 c	14.33 c	11.81 c	6.14 c	84.06 c
**5**	36.71 b	53.59 b	30.72 b	17.83 b	15.36 b	9.51 b	86.09 b
**10**	39.50 a	60.29 a	37.15 a	23.32 a	20.96 a	14.62 a	89.29 a
**LSD 0.05**	**2.91**	**4.56**	**5.1**	**2.31**	**3.98**	**3.51**	**1.90**

Means with different letters significantly differ from each other (p<0.05). Here, N_leaves_ = number of leaves per plant, SFW = shoot fresh weight, RFW = root fresh weight, SDW = shoot dry weight, RDW = root dry weight, RWC = relative water contents, NS = non-significant

Increasing NPK dose linearly improved all measured traits of Juniper tree during the study. The highest values of tree height, number of leaves per plant, fresh and dry weights of stem and root, and relative water contents were recorded for the trees sprayed with 10 g/L NPK solution, whereas the lowest values of these traits were recorded for the plants receiving no NPK throughout the growing season (Table 3).

**Fig 1:**
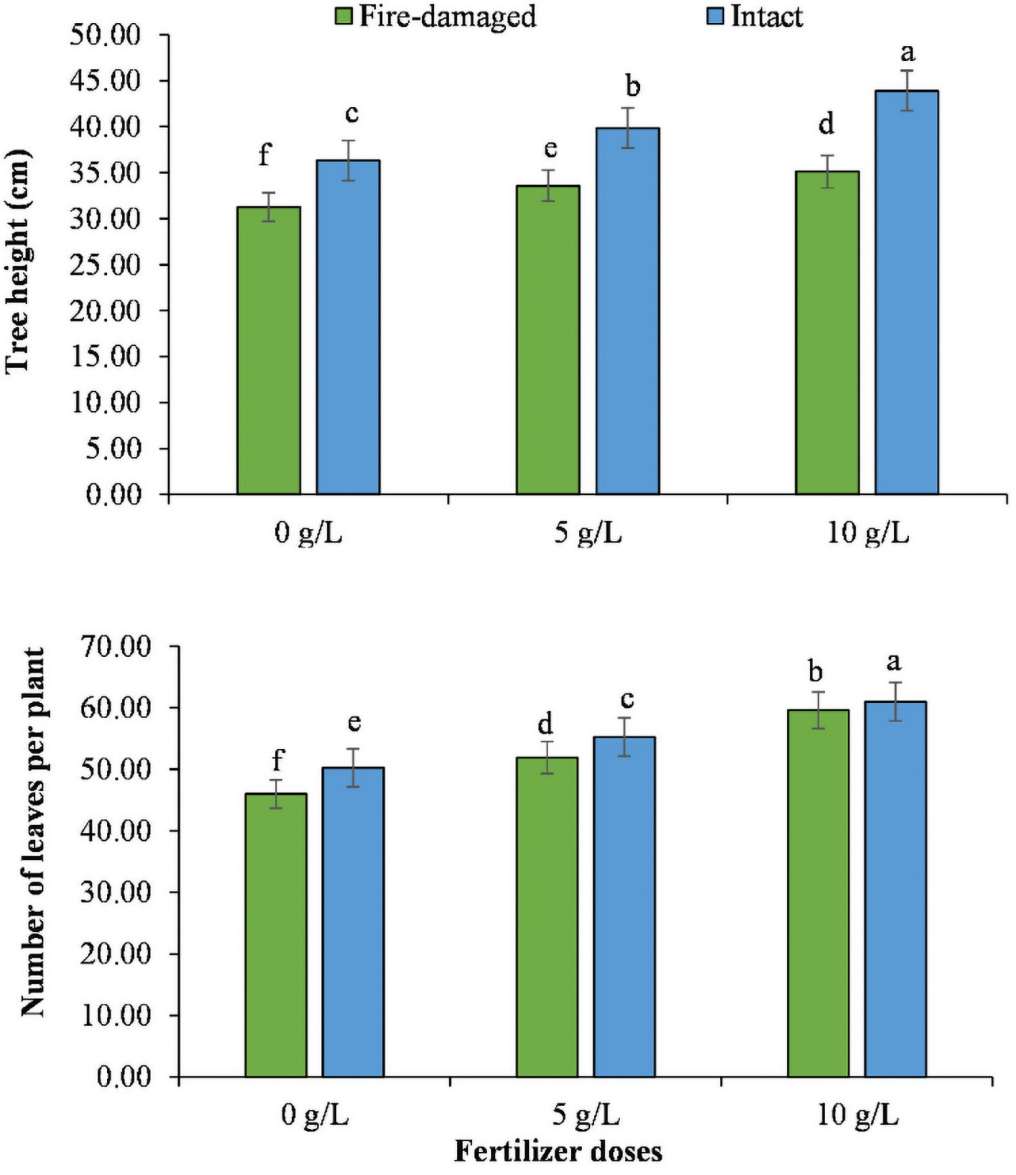
The impact of different NPK doses on tree height and number of leaves per plant of Juniper tree grown on fire-damaged and non-damaged soils.

**Fig 2:**
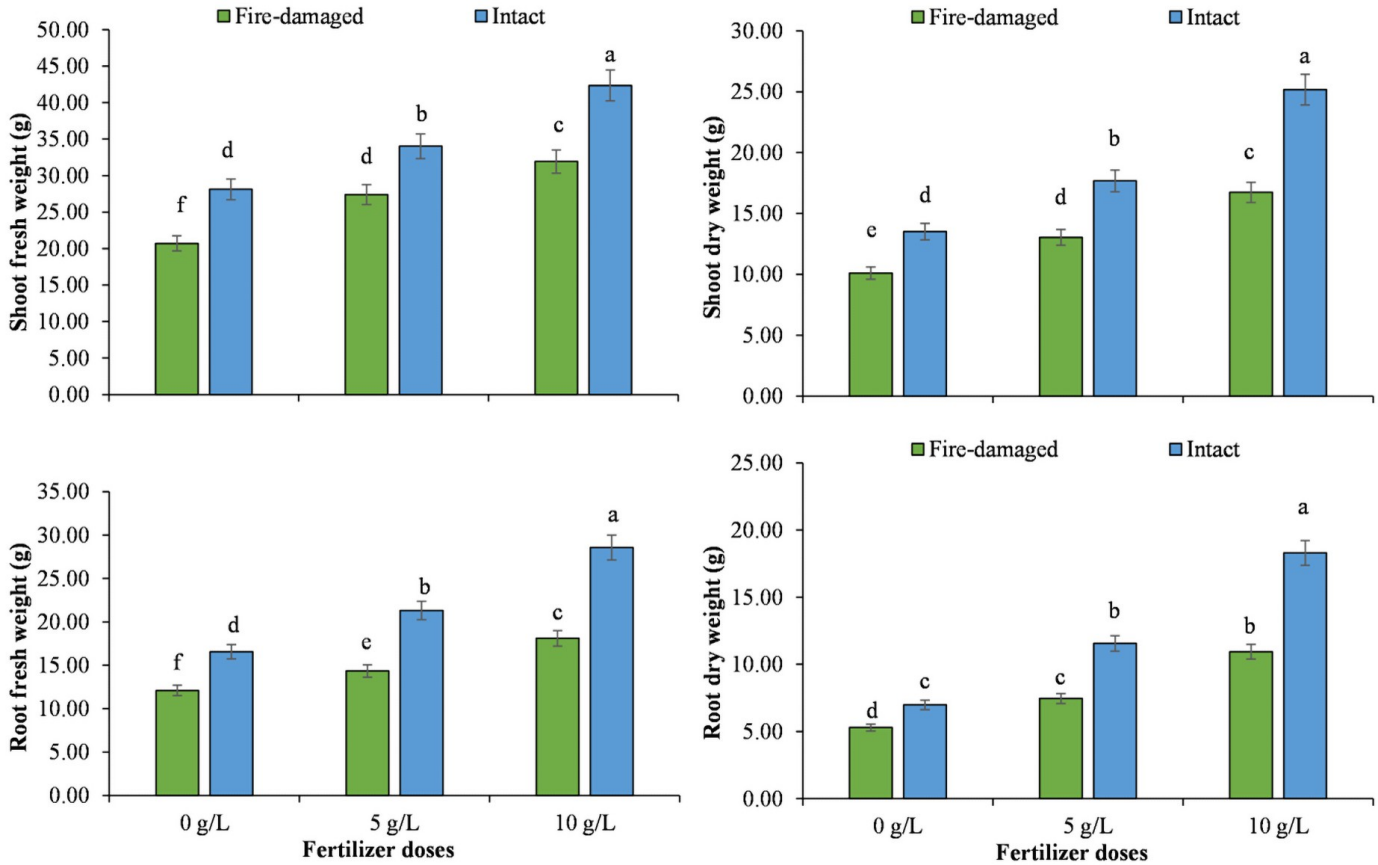
The impact of different NPK doses on fresh and dry biomass accumulation in roots and shoots of Juniper tree grown on fire-damaged and non-damaged soils.

**Fig 3:**
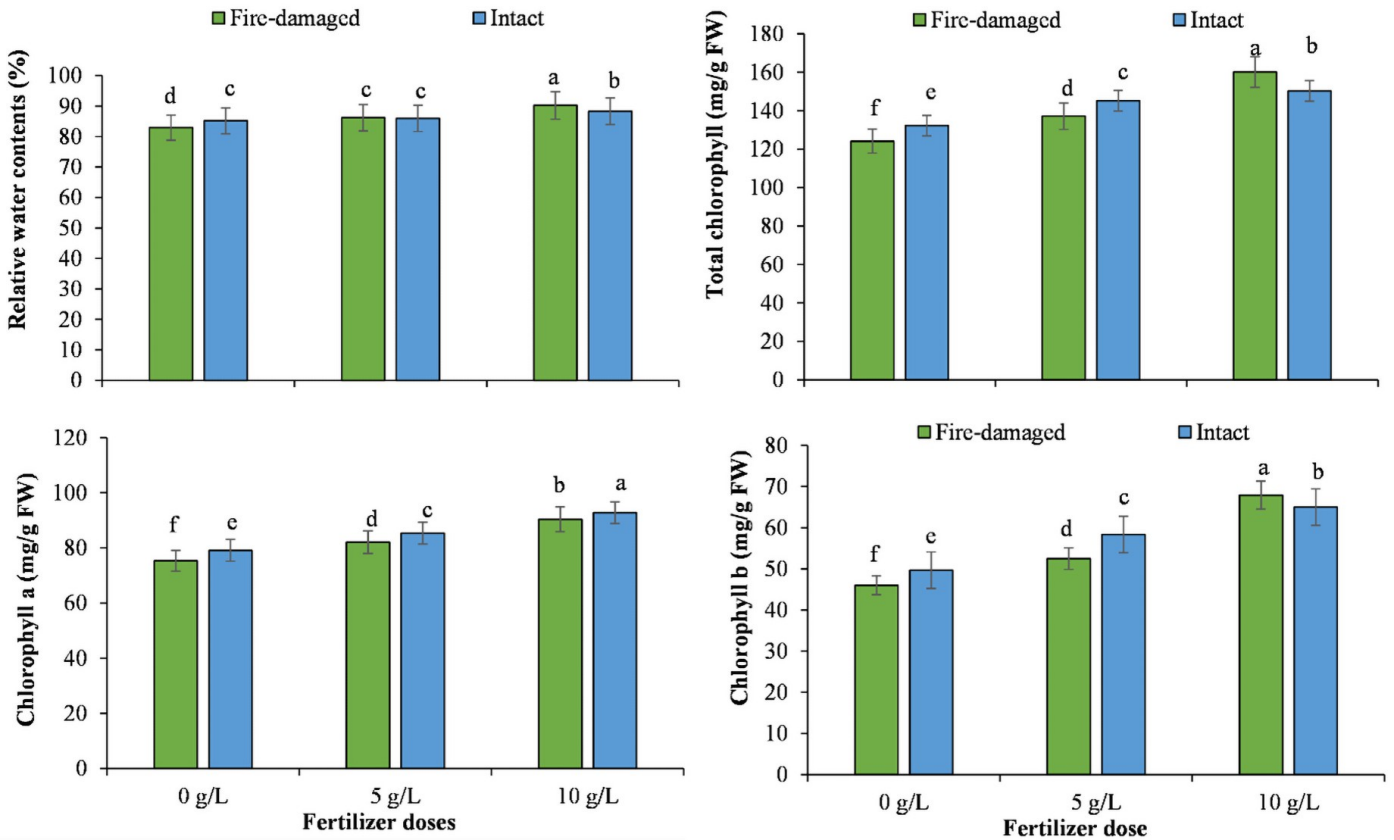
The impact of different NPK doses on relative water and chlorophyll (a, b and total) contents of Juniper tree grown on fire-damaged and non-damaged soils.

Regarding soil types by NPK concentrations interaction, higher values for tree height, number of leaves per plant, fresh and dry weights of stem and root, and relative water contents were recorded for the trees grown on intact soil with 10 g/L NPK, whereas the lowest values for these traits were noted for the trees grown on fire-damaged soil with 0 g/L NPK (Figs 1–3).

The individual and interactive effects of soil types and NPK concentrations significantly altered chlorophyll a, chlorophyll b, total chlorophyll, accumulation of nitrogen, phosphorus, potassium, sodium, and potassium sodium ratio of *J*. *procera* tree with some exceptions (Table 4). Soil type had the only non-significant impact on potassium sodium ratio, whereas the rest of the traits significantly affected all the measured biochemical and nutrient acquisition traits during the study (Table 4).

**Table 4:** Analysis of variance of different soil types and NPK doses for chlorophyll contents and nutrient acquisition traits of juniper tree.

Source of variation	DF	Sum of squares	Mean squares	F value	P value
**Chlorophyll a**					
**Soil Type (S)**	1	44.94	44.94	152.81	< 0.0001*
**Nitrogen doses (N)**	2	621.22	310.61	1056.28	< 0.0001*
**S × N**	2	1.48	0.74	2.52	0.1220^NS^
**Chlorophyll b**					
**Soil Type (S)**	1	21.91	21.91	67.26	< 0.0001*
**Nitrogen doses (N)**	2	1052.11	526.05	1614.79	< 0.0001*
**S × N**	2	62.83	31.42	96.44	< 0.0001*
**Total chlorophyll**					
**Soil Type (S)**	1	20.14	20.14	42.21	< 0.0001*
**Nitrogen doses (N)**	2	2180.13	1090.07	2284.35	< 0.0001*
**S × N**	2	324.48	162.24	339.99	< 0.0001*
**Nitrogen accumulation**					
**Soil Type (S)**	1	83.51	83.51	643.51	< 0.0001*
**Nitrogen doses (N)**	2	241.32	120.66	929.83	< 0.0001*
**S × N**	2	17.56	8.78	67.65	< 0.0001*
**Phosphorus accumulation**					
**Soil Type (S)**	1	61.09	61.09	158.31	< 0.0001*
**Nitrogen doses (N)**	2	400.86	200.43	519.42	< 0.0001*
**S × N**	2	2.75	1.37	3.56	0.0610 ^NS^
**Potassium accumulation**					
**Soil Type (S)**	1	79.13	79.13	315.94	< 0.0001*
**Nitrogen doses (N)**	2	171.62	85.81	342.63	< 0.0001*
**S × N**	2	4.27	2.13	8.52	0.0050*
**Sodium accumulation**					
**Soil Type (S)**	1	10.16	10.16	160.40	< 0.0001*
**Nitrogen doses (N)**	2	12.12	6.06	95.74	< 0.0001*
**S × N**	2	3.81	1.91	30.10	< 0.0001*
**Potassium Sodium ratio**					
**Soil Type (S)**	1	0.37	0.37	7.88	0.0159 ^NS^
**Nitrogen doses (N)**	2	27.82	13.91	299.35	< 0.0001*
**S × N**	2	0.41	0.20	4.41	0.0367*

* = significant,

NS = non-significant, DF = degree of freedom

**Fig 4:**
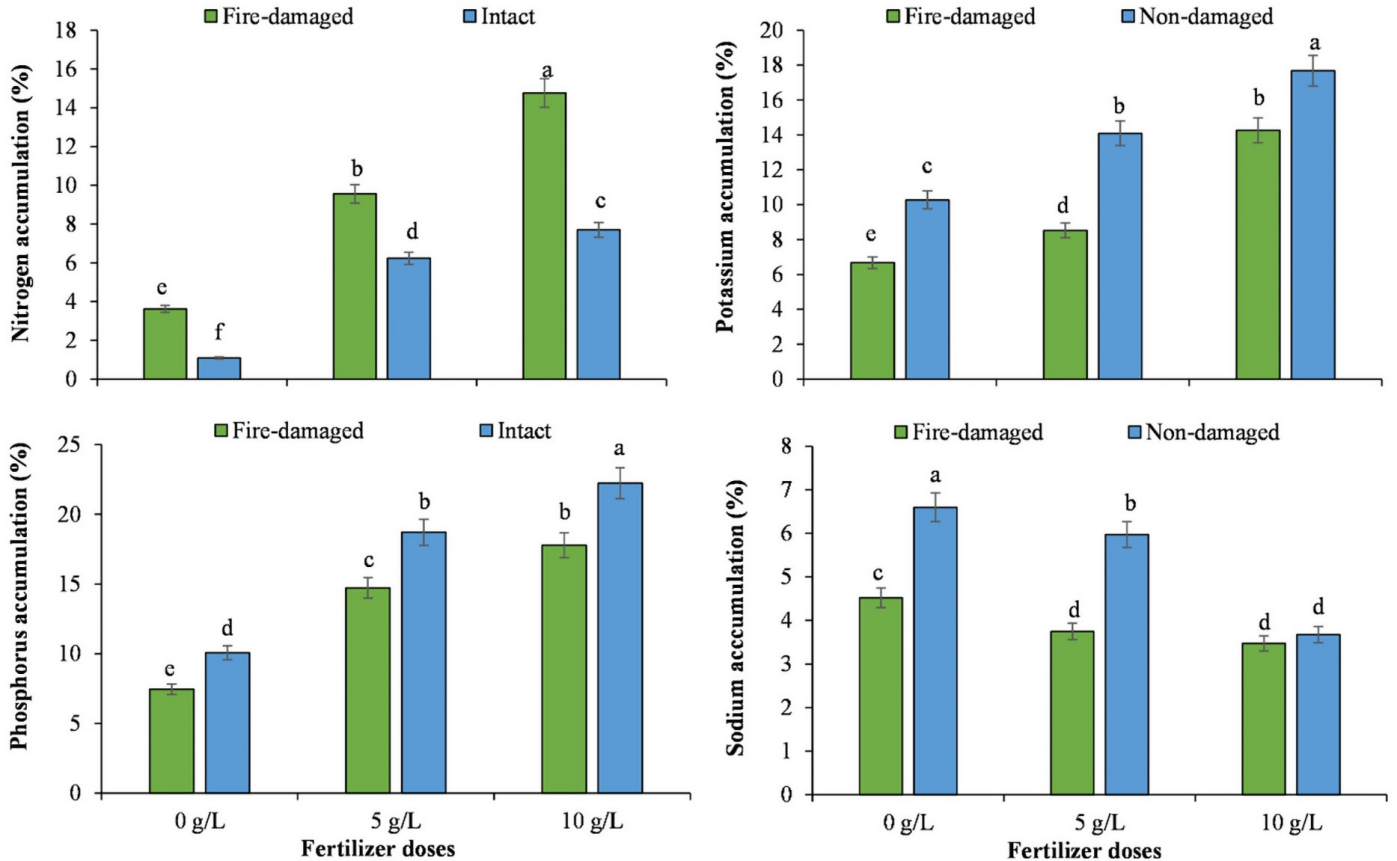
The impact of different NPK doses on nutrient accumulation of Juniper tree grown on fire-damaged and non-damaged soils.

**Fig 5:**
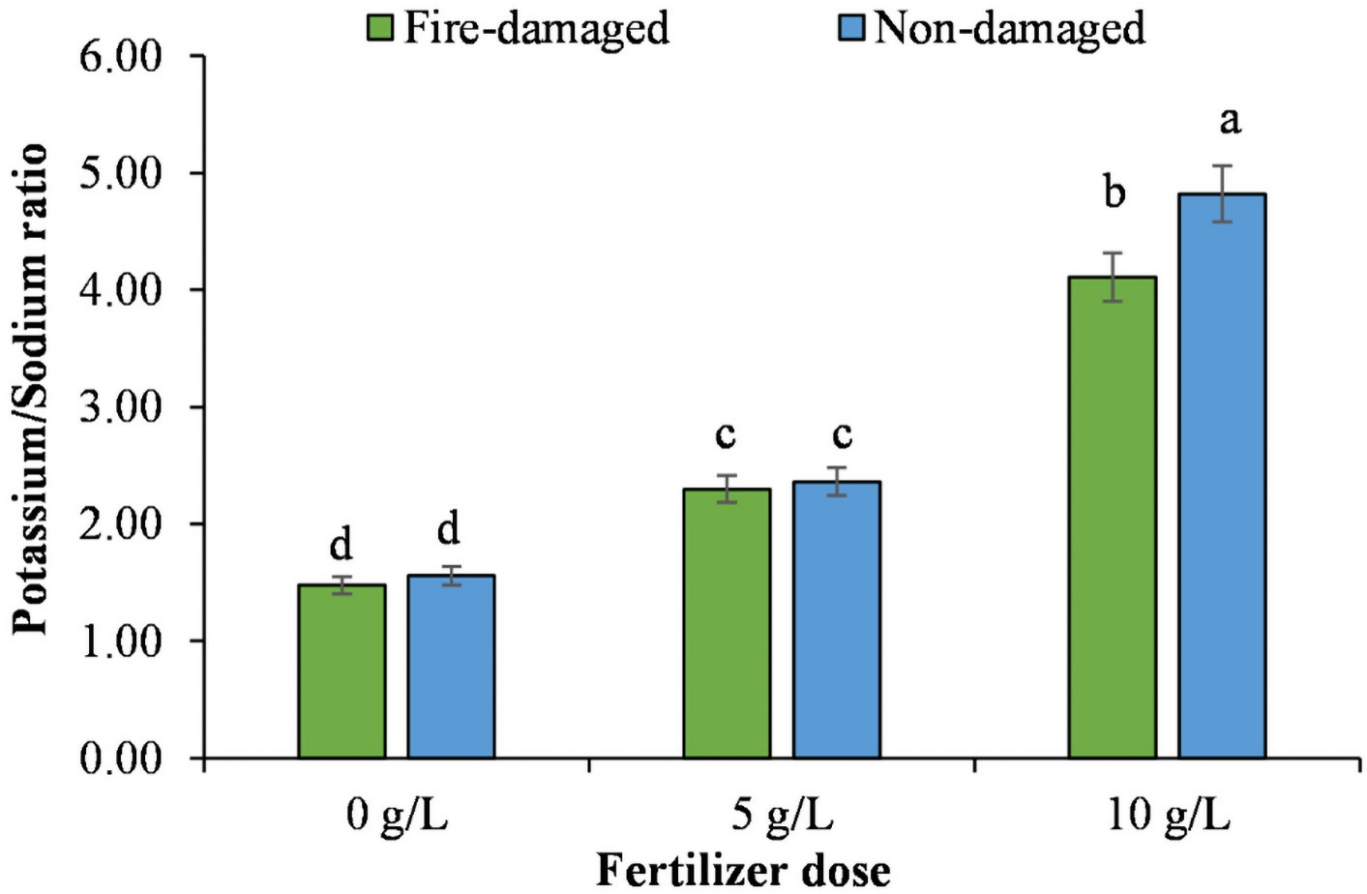
The impact of different NPK doses on potassium sodium ratio of Juniper tree grown on fire-damaged and non-damaged soils.

Regarding soil types by NPK concentrations’ interaction, higher values for chlorophyll a, chlorophyll b, total chlorophyll, phosphorus, potassium and sodium accumulation, and potassium sodium ratio were recorded for the trees grown on non-damaged soil with 10 g/L NPK, whereas the lowest values for these traits were noted for the trees grown on fire-damaged soil with 0 g/L NPK (Figs 3–5). However, tress grown on fire-damaged soil with the highest NPK concentration recorded the highest nitrogen accumulation compared to the rest of the treatments included in the study (Fig 4).

## Discussion

The individual and interactive effects of soil types and NPK doses significantly altered growth and biochemical attributes and nutrient accumulation in Juniper tree as hypothesized. The lowest values for these traits were noted for the trees grown on fire damaged soil with no NPK application, whereas application of 10 g/L NPK on intact soil resulted in the highest values for these traits. Interestingly, N uptake was increased in fire-damaged soil compared to intact soil. Nitrogen is considered as the most limiting factor for plant growth, and it was the most deficit in fire-damaged soil (Table 1). Similarly, the P and K concentration in the fire-damaged soil was lower than the intact soil. Nevertheless, the intact soil has higher C and N stocks compare to the fire-damaged soils. Several earlier studies have reported that wildfires significantly lower nutrient concentration in the soils [7, 9]. The application of NPK increased the availability of nutrients in both soil types which enabled Juniper trees to extract the nutrient and improve their growth. The higher values of observed traits on non-damaged soil under the highest NPK concentration are directly linked with the continuous supply of essential nutrients throughout the growth period which resulted in better tree growth.

Plants exploit nutrient acquisition to persist in different habitats and higher acquisition of nutrients results in better growth [47]. Similar results were obtained in the current study where plants with higher nutrient acquisition resulted in better growth. The nutrient acquisition ability of plant species considerably varies depending upon the nature of species and habitat where they are being established [47]. Successful restoration of forests can be achieved by selecting the species with better nutrient acquisition traits [48].

Plant species possess numerous growth traits which enable them to persist in various ecosystems [49]. Higher nutrient acquisition is among the most important traits of tree species enabling them to persist under various environments [50]. Juniper trees grown with the highest NPK concentration achieved higher nutrients, which enabled them to successfully grow on both soil types. The growth of the tress was significantly increased with increasing NPK levels on fire-damages soils indicating that addition of NPK could accelerate the growth of Juniper trees on damaged soils. Higher growth and biomass accumulation are considered important traits for the successful establishment of plants [51]. Tree height is another important trait as taller trees could produce more offspring compared to the trees having shorter height [47].

Nitrogen (N), phosphorus (P) and potassium (K) are primary nutrients required by the plants for their proper growth and development. Growth and development of plants is negatively affected by the deficiency of any of these nutrients during their life cycle. Nitrogen plays a key role chlorophyll synthesis and subsequently in photosynthesis. Nitrogen plays an important role in vegetative growth of plants; thus, should remain available throughout this stage [52]. The P increases cell division and stimulates root growth and flowering. The P is found in the plant parts having high metabolism and rapid cell division; therefore, plays a role in the storage and transfer of energy released during photosynthesis and its deficiency delays plant maturity [26]. Rational/artificial fertilization could improve soil nutrient status which would increase crop productivity and alter C and N input from crop residues. The fertilization also influences soil C and N accumulation through change on soil structure, soil organic C and N component and soil aggregation [27]. However, optimum amount of these nutrients to be supplied should be determined prior to their application.

## Conclusions

Increasing NPK doses significantly increased growth and biochemical attributes and nutrient acquisition of *Juniperus procera* grown on fire-damaged and intact soils. The highest values of these traits were recorded with 10 g/L NPK spray. Hence, 10 g/L NPK can be used to improve plant growth and establishment in fire affected areas of Asir province, Saudi Arabia.

## Supporting information

S1 File Minimal dataset of the study used to build graphs and analyze the data reported and interpreted in the manuscript. (XLSX)

## References

[1] El-Juhany LI, Aref IM, Al-Ghamdi MA. The possibility of ameliorating the regeneration of juniper trees in the natural forests of Saudi Arabia. Res J Agric Biol Sci. 2008;4: 126–133.

[2] Adams RP. Junipers of the world: the genus Juniperus. Trafford Publishing; 2014.

[3] Al-Ghamdi AAM, Jais HM. Interaction between Arbuscular Mycorrhiza and Heavy Metals in the Rhizosphere and Roots of Juniperus procera. Int J Agric Biol. 2012;14.

[4] AL-Ghamdi AAM, Jais HM, Khogali A. Relationship between the status of arbuscular mycorrhizal colonization in the roots and heavy metal and flavonoid contents in the leaves of Juniperus procera. J Ecol Nat Environ. 2011;4: 212–218.

[5] Jain TB, Gould WA, Graham RT, Pilliod DS, Lentile LB, González G. A soil burn severity index for understanding soil-fire relations in tropical forests. Ambio. 2008. doi: 10.1579/0044-7447-37.7.563 19205179

[6] Ne’eman G, Izhaki I, Keeley JE. Fire in the Mediterranean—from genes to ecosystems. Isr J Ecol Evol. 2012;58: 103–111.

[7] Jhariya MK, Singh L. Effect of fire severity on soil properties in a seasonally dry forest ecosystem of Central India. Int J Environ Sci Technol. 2021. doi: 10.1007/s13762-020-03062-8

[8] Santín C, Doerr SH. Fire effects on soils: The human dimension. Philosophical Transactions of the Royal Society B: Biological Sciences. 2016. 10.1098/rstb.2015.0171 PMC4874409 27216528

[9] Singh AK, Kushwaha M, Rai A, Singh N. Changes in soil microbial response across year following a wildfire in tropical dry forest. For Ecol Manage. 2017. doi: 10.1016/j.foreco.2017.02.042

[10] Alcasena FJ, Ager AA, Salis M, Day MA, Vega-Garcia C. Optimizing prescribed fire allocation for managing fire risk in central Catalonia. Sci Total Environ. 2018. doi: 10.1016/j.scitotenv.2017.11.297 29216595

[11] Page SE, Hooijer A. In the line of fire: The peatlands of Southeast Asia. Philosophical Transactions of the Royal Society B: Biological Sciences. 2016. 10.1098/rstb.2015.0176 PMC4874413 27216508

[12] Zhan Y, Liu F, Peng X, Wang G. The effects of different burning intensities on soil properties during recovery stage of forests in subtropical China. J Soil Water Conserv. 2020. doi: 10.2489/JSWC.75.2.166

[13] Jhariya MK, Singh L. Herbaceous diversity and biomass under different fire regimes in a seasonally dry forest ecosystem. Environ Dev Sustain. 2021. doi: 10.1007/s10668-020-00892-x

[14] Doblas-Miranda E, Martínez-Vilalta J, Lloret F, Álvarez A, Ávila A, Bonet FJ, et al. Reassessing global change research priorities in mediterranean terrestrial ecosystems: How far have we come and where do we go from here? Glob Ecol Biogeogr. 2015. doi: 10.1111/geb.12224

[15] Moreira F, Viedma O, Arianoutsou M, Curt T, Koutsias N, Rigolot E, et al. Landscape—wildfire interactions in southern Europe: Implications for landscape management. Journal of Environmental Management. 2011. doi: 10.1016/j.jenvman.2011.06.028 21741757

[16] Pausas JG, Llovet J, Rodrigo A, Vallejo R. Are wildfires a disaster in the Mediterranean basin? A review. International Journal of Wildland Fire. 2008. doi: 10.1071/WF07151

[17] Pausas JG, Paula S. Fuel shapes the fire-climate relationship: Evidence from Mediterranean ecosystems. Glob Ecol Biogeogr. 2012. doi: 10.1111/j.1466-8238.2012.00769.x

[18] Pausas JG, Fernández-Muñoz S. Fire regime changes in the Western Mediterranean Basin: From fuel-limited to drought-driven fire regime. Clim Change. 2012. doi: 10.1007/s10584-011-0060-6

[19] Puerta-Piñero C, Espelta JM, Sánchez-Humanes B, Rodrigo A, Coll L, Brotons L. History matters: Previous land use changes determine post-fire vegetation recovery in forested Mediterranean landscapes. For Ecol Manage. 2012. doi: 10.1016/j.foreco.2012.05.020

[20] Torres I, Pérez B, Quesada J, Viedma O, Moreno JM. Forest shifts induced by fire and management legacies in a Pinus pinaster woodland. For Ecol Manage. 2016. doi: 10.1016/j.foreco.2015.11.027

[21] Taboada A, Tárrega R, Marcos E, Valbuena L, Suárez-Seoane S, Calvo L. Fire recurrence and emergency post-fire management influence seedling recruitment and growth by altering plant interactions in fire-prone ecosystems. For Ecol Manage. 2017. doi: 10.1016/j.foreco.2017.07.029

[22] Verma S, Jayakumar S. Impact of forest fire on physical, chemical and biological properties of soil: A review. Proc Int Acad Ecol Environ Sci. 2012;2: 168.

[23] Duarah I, Deka M, Saikia N, Deka Boruah HP. Phosphate solubilizers enhance NPK fertilizer use efficiency in rice and legume cultivation. 3 Biotech. 2011;1: 227–238. doi: 10.1007/s13205-011-0028-2 22558541 PMC3339586

[24] Venkatesh MS, Hazra KK, Ghosh PK, Khuswah BL, Ganeshamurthy AN, Ali M, et al. Long–term effect of crop rotation and nutrient management on soil–plant nutrient cycling and nutrient budgeting in Indo–Gangetic plains of India. Arch Agron Soil Sci. 2017;63: 2007–2022. doi: 10.1080/03650340.2017.1320392

[25] Khan MB, Rafiq R, Hussain M, Farooq M, Jabran K. Ridge sowing improves root system, phosphorus uptake, growth and yield of Maize (Zea Mays L.) Hybrids. J Anim Plant Sci. 2012;22: 309–317.

[26] Savoy H. Fertilizers and their use. PB-1637, Agric Ext Serv Univ Tennessee, Knoxv. 1999.

[27] Su YZ, Zhao WZ, Su PX, Zhang ZH, Wang T, Ram R. Ecological effects of desertification control and desertified land reclamation in an oasis–desert ecotone in an arid region: a case study in Hexi Corridor, northwest China. Ecol Eng. 2007;29: 117–124.

[28] Hai L, Li XG, Li FM, Suo DR, Guggenberger G. Long-term fertilization and manuring effects on physically-separated soil organic matter pools under a wheat–wheat–maize cropping system in an arid region of China. Soil Biol Biochem. 2010;42: 253–259.

[29] Andersson F, Lhoir P. Introduction. Conifers. Ecosyst World. 2006;6: 1–22.

[30] Negash L. Indigenous trees of Ethiopia. Biol uses Propag Tech Repro SLU, Umeå, Sweden. 1995.

[31] AL-Ghamdi AAM, Jais HM. Interaction between soil textural components, flavonoids in the roots and mycorrhizal colonization in Juniperus procera in Saudi Arabia. African J Microbiol Res. 2013;7: 996–1001.

[32] El Atta HA, Aref I. Effect of terracing on rainwater harvesting and growth of Juniperus procera Hochst. ex Endlicher. Int J Environ Sci Technol. 2010;7: 59–66.

[33] Khoshhal Sarmast M, Mosavizadeh SJ, Sharifani M. Evaluation of Junipers spp. Genetic Diversity in Northern Iran using ISSR Markers. Ecol Iran For. 2018.

[34] Ghorbanzadeh A, Ghasemnezhad A, Sarmast MK, Ebrahimi SN. An analysis of variations in morphological characteristics, essential oil content, and genetic sequencing among and within major Iranian Juniper (Juniperus spp.) populations. Phytochemistry. 2021. doi: 10.1016/j.phytochem.2021.112737 33740576

[35] Nelson DW, Sommers LE. Total carbon, organic carbon, and organic matter. Methods soil Anal Part 3 Chem methods. 1996;5: 961–1010.

[36] Gee GW, Bauder JW. Particle‐size analysis. Methods soil Anal Part 1 Phys Mineral methods. 1986;5: 383–411.

[37] Rhoades JD. Cation exchange capacity. Methods Soil Anal Part 2 Chem Microbiol Prop. 1983;9: 149–157.

[38] Olsen SR. Estimation of available phosphorus in soils by extraction with sodium bicarbonate. US Department of Agriculture; 1954.

[39] Carson PL. Recommended potassium test. Bull Dep Agric Econ ND Agric Exp Stn ND State Univ Agric Appl Sci. 1975.

[40] Bremner JM. Determination of nitrogen in soil by the Kjeldahl method. J Agric Sci. 1960;55: 11–33.

[41] Arnon DI. Copper enzymes in isolated chloroplasts. Polyphenoloxidase in Beta vulgaris. Plant Physiol. 1949;24: 1. doi: 10.1104/pp.24.1.1 16654194 PMC437905

[42] Farooq S, Shahid M, Khan MB, Hussain M, Farooq M. Improving the productivity of bread wheat by good management practices under terminal drought. J Agron Crop Sci. 2015;201: 173–188. doi: 10.1111/jac.12093

[43] Onen H, Farooq S, Gunal H, Ozaslan C, Erdem H. Higher Tolerance to Abiotic Stresses and Soil Types May Accelerate Common Ragweed (Ambrosia artemisiifolia) Invasion. Weed Sci. 2017;65: 115–127. doi: 10.1614/WS-D-16-00011.1

[44] Shapiro SS, Wilk MB. An analysis of variance test for normality (complete samples). Biometrika. 1965;52: 591–611.

[45] Steel R., Torrei J, Dickey D. Principles and Procedures of Statistics A Biometrical Approach. A Biometrical Approach. 1997.

[46] IBM C, IBM SPSS Inc. SPSS Statistics for Windows. IBM Corp Released 2012. 2012;Version 20: 1–8.

[47] Gioria M, Osborne BA. Resource competition in plant invasions: emerging patterns and research needs. Front Plant Sci. 2014;5. doi: 10.3389/fpls.2014.00501 25324851 PMC4179379

[48] Lundgren MR, Small CJ, Dreyer GD. Influence of Land Use and Site Characteristics on Invasive Plant Abundance in the Quinebaug Highlands of Southern New England. Northeast Nat. 2004;11: 313–332. doi: 10.1656/1092-6194(2004)011[0313:IOLUAS]2.0.CO;2

[49] Van Kleunen M, Dawson W, Maurel N. Characteristics of successful alien plants. Mol Ecol. 2015;24: 1954–1968. doi: 10.1111/mec.13013 25421056

[50] Van Kleunen M, Weber E, Fischer M. A meta-analysis of trait differences between invasive and non-invasive plant species. Ecology Letters. 2010. pp. 235–245. doi: 10.1111/j.1461-0248.2009.01418.x 20002494

[51] van Kleunen M, Dawson W, Dostal P. Research on invasive-plant traits tells us a lot. Trends in Ecology and Evolution. 2011. p. 317. doi: 10.1016/j.tree.2011.03.019 21497938

[52] Rumeu B, Caujapé-Castells J, Blanco-Pastor JL, Jaén-Molina R, Nogales M, Elias RB, et al. The colonization history of Juniperus brevifolia (Cupressaceae) in the Azores islands. PLoS One. 2011. doi: 10.1371/journal.pone.0027697 22110727 PMC3218011

