# Correction: The impact of NPK fertilizer on growth and nutrient accumulation in juniper (*Juniperus procera*) trees grown on fire-damaged and intact soils

**DOI:** 10.1371/journal.pone.0293768

**Published:** 2023-10-26

**Authors:** Ahlam Khalofah, Hamed A. Ghramh, Rahmah N. Al-Qthanin, Boullbaba L’taief

There are errors in the Funding statement. The correct Funding statement is as follows: This work was supported by Institute of Research and Consulting Studies, King Khalid University, Saudi Arabia. The funders had no role in study design, data collection and analysis, decision to publish, or preparation of the manuscript.

## References

[1] Khalofah A, Ghramh HA, Al-Qthanin RN, L’taief B (2022) The impact of NPK fertilizer on growth and nutrient accumulation in juniper (*Juniperus procera*) trees grown on fire-damaged and intact soils. PLoS ONE 17(1): e0262685. 10.1371/journal.pone.0262685 35085316 PMC8794100

